# Accounting for the Local Field When Determining the Dielectric Loss Spectra of Metals in the Region of the Frequencies of Volume, Surface and Localized Plasmon Oscillations

**DOI:** 10.3390/ma13030631

**Published:** 2020-01-31

**Authors:** Tatiana Perova, Igor Shaganov, Kevin Berwick

**Affiliations:** 1 Department of Electronic and Electrical Engineering, Trinity College Dublin, The University of Dublin, 2 Dublin, Ireland; 2 Vavilov State Optical Institute, 199034 St.-Petersburg, Russia; shaganov.ii@gmail.com; 3 School of Electrical and Electronic Engineering, Technical University Dublin, 8 Dublin, Ireland; kevin.berwick@TUDiblin.ie

**Keywords:** metal nanoparticles, volume plasmons, surface plasmons, localized surface plasmons, solid colloids, dispersive local field approach

## Abstract

The optical constant of bulk metal is used to determine the dispersion of the local field under one-dimensional (1D), two-dimensional (2D) and three-dimensional (3D) confinement. 3D confinement, expressed as $\varepsilon_{2}^{mic}\left(\omega_{3D}\right)$, corresponds to the dielectric loss spectra of spherical particles with a diameter, *d*, much less than the wavelength of the beam used to measure the spectrum (*d* << λ). Excellent agreement with the results of Mie theory and experimental data for solid colloids within alkali halide crystals was observed. The function expressed as $\varepsilon_{2}^{mic}\left(\omega_{1D}\right)$ allows the measurement of spectral micro-characteristics in the frequency range of the longitudinal collective motion of the free electrons. This corresponds to the spectrum of dielectric losses of bulk plasma oscillations. The function $\varepsilon_{2}^{mic}\left(\omega_{2D}\right)$ describes the spectra of the dielectric losses of surface plasma oscillations in thin metal films. It is shown that the peak positions of $\varepsilon_{2}^{mic}\left(\omega_{3D}\right)$, $\varepsilon_{2}^{mic}\left(\omega_{2D}\right)$ and $\varepsilon_{2}^{mic}\left(\omega_{1D}\right)$ spectra for simple metals, viz. alkali metals as well as Al, Be, Mg, Ga, In, Sn and Si, are in agreement with experimental results from electron-energy-loss spectroscopy and various optical techniques.

## 1. Theoretical Considerations

### 1.1. Intermolecular Interactions in a Condensed Medium

Intermolecular interactions (IMIs) play an important role in the determination of the physical properties of various condensed media, their nanoparticles and composites. Studies of the spectral characteristics of these media, both in bulk and in the form of thin films and the corresponding nanocomposites, play an important role in engineering materials with unique optical properties. A targeted search for promising optical media should be informed by a deep understanding of the characteristics of their interactions with light at the microscopic level. Naturally, this necessitates a more detailed analysis of the influence of the IMIs on the optical characteristics of these media. IMIs are a consequence of the universal van der Waals interactions that exist in any condensed medium, as well as specific intermolecular interactions such as, for example, a hydrogen bond. We exclude from consideration the specific IMIs that manifest themselves in liquids and solutions of polar molecules. When considering solid media, crystals, dielectrics and metals, we confine ourselves to the classical oscillator model and the dipole approximation, according to which a harmonic oscillator or dipole is compared to each energy level of a condensed molecular medium. The interaction of these dipoles at the resonant frequency is considered. The basis of the experimentally recorded response of a condensed medium to the electromagnetic field of the optical frequency, *E*(*ν*), is a result of averaging the elementary interactions of its constituent atoms, or molecules, with a local effective field of the light wave *E_eff_*(*ν*) [1,2].

The role of the local field in IMI spectroscopy can be clearly understood based on the formation of the Hamiltonian (H), the energy operator of the condensed medium. Considering the interaction between the molecules of the medium, the IMI potential of the medium, to a first approximation, can be described as two additions to the operator of the energy of noninteracting molecules:

H = H_0_ + H_1_ + H_2_, (1)

where the operator H_1_ characterizes the contribution of the external field *E*(*ω*), considered as a weak perturbation. In the dipole approximation, this contribution is defined as H_1_ = *µE_eff_*(*ω*), where *µ* is the matrix element of the dipole moment of the energy transition under consideration. The probability of the transition, in accordance with Einstein’s expression, is determined by the square of this dipole moment as

(2) $$B_{jk}=\left(8\pi^{3}/3h^{2}\right){\left|\mu_{jk}\right|}^{2},$$

where *B_jk_* is the Einstein integral coefficient, which determines the total probability of transition *j–k* with a unit integral density of the effective field *u_eff_* in accordance with the following expression [3]

α_jk_ = *B_jk_u_eff_*.

The third term in Equation (1), $H_{2}=\mu {\left|E_{eff}\left(\nu \right)\right|}^{2}$, takes into account the change in the potential of the IMI forces due to the nonlinear effects observed in powerful optical fields. In this paper, we consider the external field *E*(*ν*) acting on a condensed medium as a weak perturbation and the influence of an IMI on the absorption spectra of condensed media will be limited to a linear optics approach. It will be shown later that the dipole term H_1_ can be represented as a sum of two terms, one of which considers the contribution of resonant dipole–dipole interactions of like oscillators, while the other takes into account the interactions of the oscillator with non-absorbing environmental molecules, known as induction–dipole interactions. Estimation of the potential of the full interaction of the molecule with the environment, considering pair potentials of dispersion, induction and resonance interactions [4], is a difficult task. It is possible to apply a simpler method to consider the IMI in general by replacing the real interaction of a given molecule with the environmental molecules with a local effective field acting upon it. This approach, developed in [5,6], has been very successful in analysing the spectral manifestations of resonant dipole–dipole interactions of various condensed media, including liquids, dielectrics and metals [7,8,9,10,11,12,13].

### 1.2. The Manifestation of the Spectral Differences of the Effective and Average Fields in a Condensed Medium

According to the ideas presented in [5,7], the spectral probability density of the absorption quantum transition *α*(*ν*) can be represented from both a micro and macro point of view in the form

*α*(*ν*) = *B*(*ν*) *u*’*_eff_* (*ν*) = *K*(*ν*) *c u_av_*(*ν*)/(*Nhν*), (3)

where ν is the wave number in cm^−1^, *B*(*ν*) is the spectral density of the specific probability of the transition in question, *u’_eff_*(*ν*) = *E_eff_*(*ν*)^2^/8π and *u_av_*(*ν*) = *E_av_*(*ν*)^2^/8π are the bulk spectral energy densities of the effective and average fields, *K*(*ν*) is the absorption coefficient in cm^−1^, *h* is Planck’s constant, *c* is the speed of light in vacuum, and *N* is the concentration of absorbing centers in cm^−3^.

Polarization of the condensed medium, accompanied by dipole–dipole interactions of the constituent molecules at resonant frequencies, means that the volume spectral densities *u’_eff_*(*ν*) and *u_av_*(*ν*), corresponding to the squares of the effective and average fields, are not equal. This is reflected in the expression, which follows from Equation (3) and reflects the relationship between the macroscopic and microscopic characteristics of the absorption resonance under consideration:

(4) B(ν)=K(ν)n(ν)c|θ(ν)|/(Nhν),

where *θ*(*ν*) is the local field factor, bearing in mind differences in the strength of the effective and mean fields at a given frequency. Since the square of the modulus of the effective field factor in the region of strong absorption bands is spectrally sharp, the spectroscopic characteristics of a condensed medium may differ significantly from the characteristics of the corresponding quantum transitions.

Expression (4) was the basis of the so-called dispersion of effective field method (DEF) [5,6], developed more than half a century ago. This method is based on a comparison of the experimental absorption spectrum of a condensed medium in the region of the absorption band and the corrected spectrum *B*(*ν*) obtained from Expression (4). The DEF method allows a visual analysis of the role of dipole–dipole interactions in distinguishing the frequencies and integrated intensities of condensed media from the characteristics of the microscopic oscillators responsible for their absorption. It was shown later that data obtained by the DEF method are in good agreement with the results of calculations performed using the theory of IMI. Subsequent studies have shown that the concepts discussed above regarding the microcharacteristics of condensed media are valid not only for individual molecular oscillators, but also for nanoparticles or clusters with characteristic sizes much shorter than the length of the probe radiation. As shown in [11], the absorption of these particles is determined by the optical characteristics of the bulk material of the medium under dielectric confinement conditions, i.e., in the absence of polarization of the medium at the frequency of the resonant absorption considered. It is more convenient to express Equation (5) in the form

(5) $$NhB\left(\nu \right)/2\pi =\varepsilon_{2}\left(\nu \right)\theta \left(\nu \right),$$

where *ε*_2_(*ν*) is the dielectric loss spectrum of the condensed medium, the imaginary part of its dielectric constant *ε*(*ν*) = *ε*_1_(*ν*) − *iε*_2_(*ν*), and $\theta \left(\nu \right)={\left|E_{av}\left(\nu \right)/E_{eff}\left(\nu \right)\right|}^{2}$ is the Lorentz polarization correction, taking into account the spectral differences of microscopic, *E_eff_*(*ν*), and macroscopic, *E_av_*(*ν*), fields in the condensed medium.

As shown in [12], the left-hand side of (5) can be considered as a microscopic component of the dielectric loss spectrum of a condensed medium. Thus, Expression (5), which relates the dielectric loss spectrum of a condensed medium and its corresponding microscopic analog, takes the form

(6) $$\varepsilon_{2}^{mic}\left(\nu \right)=\varepsilon_{2}\left(\nu \right)\theta \left(\nu \right).$$

This method shows good agreement between the frequencies of the maxima of the $\varepsilon_{2}^{mic}\left(\nu \right)$ spectra and the natural frequencies of vibration of an alkali halide crystal lattice, calculated both from their elastic characteristics and observation of the absorption spectra of their microcrystals [9]. For three-dimensional confinement, the $\varepsilon_{2}^{mic}\left(\nu \right)$ spectrum characterizes the dielectric loss spectrum of an isolated spherical crystal particle, with dimensions much smaller than the wavelength of the probe radiation. Similar results were obtained for nanoparticles of noble metals [13]. Therefore, Equation (6) corresponds to the spectral characteristics of an isotropic micro-object of size *d* satisfying the conditions *a_molec_* << *d* << λ, where *a_molec_* is the size of molecules and *d* is the size of so-called mesomolecules (microregions of the system under consideration) [12,14].

### 1.3. Consideration of the Differences between the Effective E_eff_(ν) and the Average E_av_(ν) Fields in a Condensed Medium

From Equations (4) and (5), the most important condition for the applicability of the approach under consideration here is a true determination of the local field factor. As shown earlier [5,6], the effective local field factor can be expressed in terms of the experimental optical characteristics (the refractive index, *n*(*ν*), and absorption index, *k*(*ν*)) using one of the models (Lorentz or Onsager) from the theory of polarisation of dielectrics. From the Lorentz model, the expression for the effective field factor for non-polar isotropic condensed matter and for two-atom cubic crystals is

(7) $${\left|\theta \left(\nu \right)\right|}^{-1/2}=\frac{\left(\varepsilon \left(\nu \right)+2\right)}{3}.$$

Equation (7) should allow calculation of the spectral characteristics of spheroidal nanoparticles of the mesophase [14], which meet the condition *d* << λ, but can still be described by the complex dielectric permittivity *ε*(*ν*). In order to confirm this, as well as to obtain expressions for other particle shapes, in Ref. [12], we deduced an equation for the effective dielectric permittivity, *ε^comp^*(*ν*), of a two-component medium containing spheroidal particles of different isotropic materials, A and B. Using a generalisation of the familiar Maxwell–Garnett expression [15,16] based on the Lorentz–Lorentz model and using expressions for the local field factor via a local field tensor from [14], we obtained a general expression for different nanoparticle shapes, with different form factors *L* and different filling factors *f* (at *f* << 1)

(8) $$\varepsilon_{2}^{comp}\left(\nu \right)=f\varepsilon_{2}^{mic}\left(\nu \right)=f\varepsilon_{2}^{bulk}\left(\nu \right)\theta_{mD}\left(\nu \right),$$

(9) $$\mathrm{where}\;\theta_{mD}\left(\nu \right)={\left|1+\frac{\left|\varepsilon \left(\nu \right)-\varepsilon_{h}\right|}{m\varepsilon_{h}}\right|}^{-2},$$

and *m* = 1, 2 and 3 corresponds to the form factor *L* = 1, 1/2 and 1/3, for 1D, 2D and 3D confinement, respectively. This, in turn, corresponds to particle shapes of a prolate spheroid, oblate spheroid and sphere, respectively. At intermediate particle shapes, the value of *m* can be varied in the range from 1 to 3.

This paper will describe calculations of the spectral optical micro-characteristics of a number of alkali metals, as well as Al, Be, Ga, In, Sn, Mg, Si and Ag for comparison. Results will be presented for 1D, 2D and 3D size confinement, together with a comparison of results with alternative theoretical descriptions and experimental data available in the literature. The metals considered were selected since their bulk plasmon resonance is located far from the interband transitions, so they won’t affect the shape and peak position of the bulk plasma oscillations at *ω_p_*. This is not the case in, for example, noble metals, where this effect is quite noticeable (see, for example, [13] and a short discussion at the end of this paper).

## 2. Results of Model Calculations

In order to easily compare the peak positions of the calculated dielectric function with the bulk and surface plasma frequencies (*ω_p_* and *ω_s_*) known from the literature, all calculations were done in units of energy (eV), i.e., ω=ℏϖ, where ϖ is the cyclic frequency and ℏ is the reduced Planck’s constant. Calculations of the spectra $\varepsilon_{2}^{mic}\left(\omega \right)$ were performed for 1D, $\varepsilon_{2}^{mic}\left(\omega_{1D}\right)$, 2D, $\varepsilon_{2}^{mic}\left(\omega_{2D}\right)$ and 3D, $\varepsilon_{2}^{mic}\left(\omega_{3D}\right)$, size confinement using Equations (8) and (9) at *f* = 0.01 and *ε_h_* = 1. Values for (*n*(*ω*) and *k*(*ω*)) of the bulk metal were taken from various references, viz. from Ref. [17] for Al and Si, from Ref. [18] for Li, Na, K and Be, from Ref. [19] for Rb, Cs and In, from Ref. [20] for Mg, from Ref. [21] for Ga and from Ref. [22] for Sn. For Ag, the optical constants were taken from Ref. [23].

**Figure 1:**
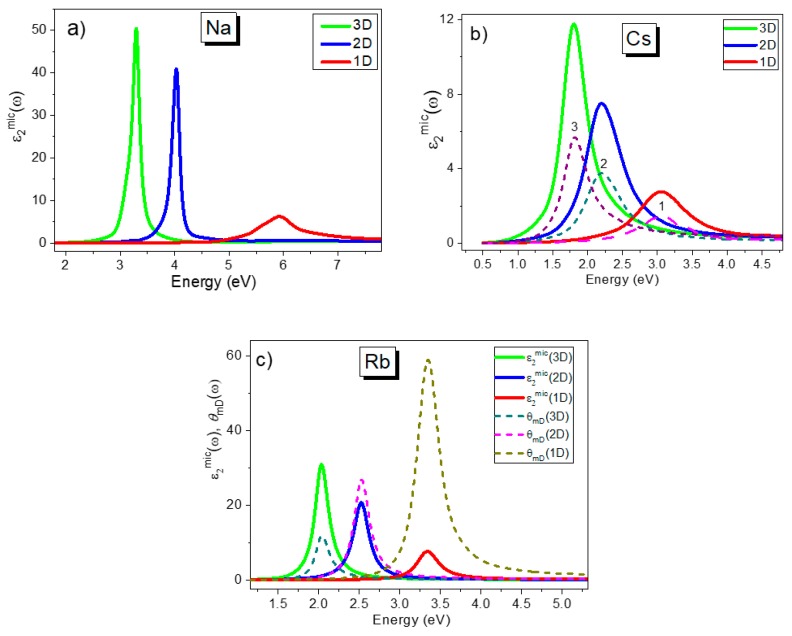
Calculated spectra $\varepsilon_{2}^{mic}\left(\omega \right)$ for 1D, 2D and 3D confinement for (**a**) Na, (**b**) Cs and (**c**) Rb. In Figure 1b, the result of calculations using Equation (11) is shown by the dashed curve 3, while dashed curve 2 corresponds to the calculated Im[1/(*ε*(*ω*) + 1)] function and the dashed curve 1 to the Im[1/*ε*(*ω*)] function. Note that the dashed curves are normalized using different factors for convenience of presentation. In (**c**), the dotted lines correspond to the frequency dependent local field factor *θ_mD_*(*ω*), calculated using Equation (9) at *m* = 1, 2 and 3.

**Figure 2:**
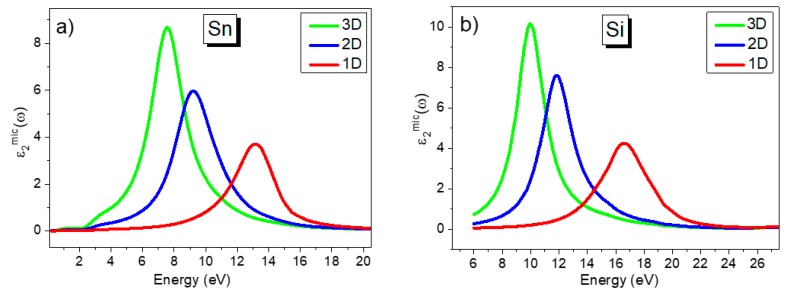

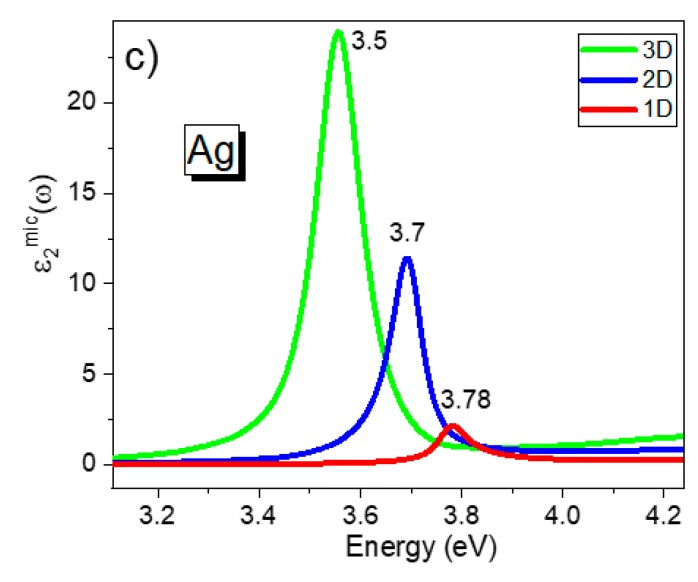
Calculated spectra $\varepsilon_{2}^{mic}\left(\omega \right)$ for 1D, 2D and 3D confinements for (**a**) Sn, (**b**) Si and (**c**) Ag.

Spectra $\varepsilon_{2}^{mic}\left(\omega_{3D}\right)$, $\varepsilon_{2}^{mic}\left(\omega_{2D}\right)$ and $\varepsilon_{2}^{mic}\left(\omega_{1D}\right)$ calculated for Na, Rb and Cs, as well as for Sn, Si and Ag, are shown in Figure 1 and Figure 2, respectively. From the figures, the peak positions of the spectra calculated for 1D, 2D and 3D dielectric confinement are significantly different for most of the simple metals considered. In order to compare the results obtained with alternative theoretical calculations and with experimental data from the literature, we consider all three cases in separate sections below.

### 2.1. Three-Dimensional Dielectric Confinement

**Figure 3:**
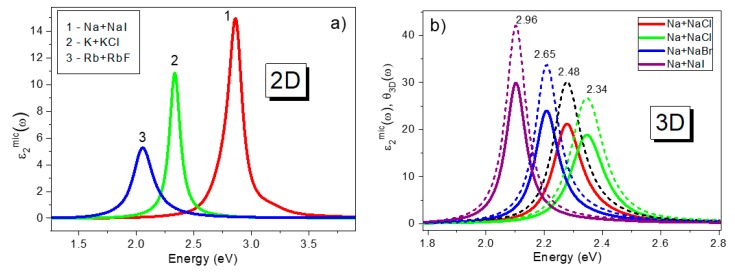

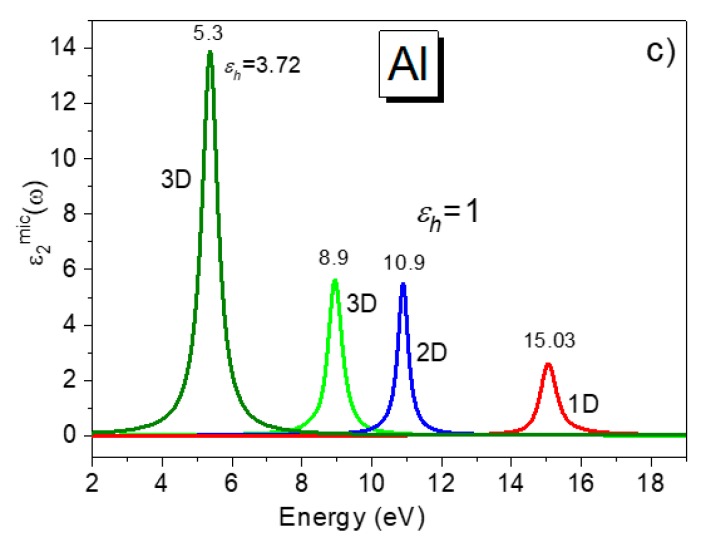
Calculated spectra $\varepsilon_{2}^{mic}\left(\omega \right)$ for (**a**) 2D confinement, shown for solid colloids Na + NaI, K + KF and Rb + RbF, (**b**) for 3D confinement, shown by solid lines for solid colloids Na + NaCl, Na + NaBr and Na + NaI corresponding to different *ε_h_* values. The dotted lines correspond to *θ_3D_*(*ω*) functions, calculated using Equation (9). (**c**) shows the results of calculations of $\varepsilon_{2}^{mic}\left(\omega \right)$ for 1D, 2D and 3D confinements for Al metal particles in air, at *ε_h_* = 1 (red, blue and green lines) and for 3D confinement for Al particles in an Al_2_O_3_ solid matrix at *ε_h_* = 3.72 (olive line).

Calculations were performed for 3D confinement of alkali metal and Al nanoparticles embedded in various media with a dielectric constant of *ε_h_*. Examples of the results obtained are shown in Figure 3. In addition, in Figure 3b, the spectral dependencies of the local field factor *θ_3D_*(*ω*) calculated with Equation (9) at *m* = 3 are also plotted by dashed lines as an example for Na metal particles in various solid matrices. The frequencies of the maxima of the spectra obtained are summarized in Table 1. Note that for these calculations the values of *ε_h_* given in column XI for alkali halides were taken from Ref. [24]. These calculations were performed in order to compare the results obtained from our model with experimental data and calculations using alternative models available in the literature for alkali metal solid colloids.

Experimental data on colloidal solutions of alkali metal particles are difficult to obtain in practice, due to their aggressive behavior in water or organic liquid solutions. This problem can be overcome if one considers data on these metal systems in so-called colloid centres (or metallic centres) of alkali metals, in particular, small alkali metal particles in alkali halide crystals [25]. The colloid absorption bands can be observed in additively coloured alkali halides using thermal and optical coagulations [24].

The optical properties of solid colloids of alkali metals were intensively investigated in the 1960s and 70s. It is known that the position of the colloid band of metal particles can be calculated using Mie’s theory [26] for the absorption of light by metal spheres [25,26,27,28]. Mie’s theory relates this absorption band to the macroscopic optical constants of the alkali metals and the dielectric constant of the host medium, *ε_h_.* These calculations were first performed by Savostianova for a Na + NaCl colloid [27] in 1930 and later for other alkali metal colloid centres in Refs. [26,28,29,30,31,32,33,34,35].

For spheroidal metal particles [36], the frequency of the localized surface plasma oscillations can be determined from the simple equation:

(10) $$\omega_{l}=\omega_{p}/\sqrt{1+(\frac{l+1}{l}\varepsilon_{h})},$$

where *l* is varied from 1 to ∞ and *ε_h_* is the dielectric constant of the surrounding medium, with *l* = 1 for spherical particles and for *l* = ∞ for the needle-like particles. For metal colloid particles in an alkali halide, calculations based on Equation (10) and experimental data obtained in Refs. [26,28,29,30,31,32,33] from Electron–Energy–Loss Spectroscopy (EELS) and optical measurements were summarized in two review articles, see Refs. [31,35]. Some of the data obtained in previous studies are listed along with our calculations in Table 1, columns V–VIII.

We note that reasonably good agreement is seen in Table 1 between our calculations for 3D confinement and data from the literature for most of the solid colloids. However, some deviations from the experimental results and calculations made for spherical particles are noticeable, specifically for K and Rb. Results obtained for these colloids are nearly equal to those calculated for *l* = ∞, or for 2D confinement from our model, which corresponds to the surface plasma oscillations of oblate spheroids (or needle-like particles) according to Tonks [33]. These results indicate that K and Rb metals coagulate into oblate spheroid-like metal nanoparticles [30,33].

Finally, the calculations performed here using the dispersive local field approach (Equations (8) and (9)), were compared with the results of Mie calculations performed by us for these metals using the same optical constants *n*(*ω*) and *k*(*ω*) used for calculations of $\varepsilon_{2}^{mic}\left(\omega_{mD}\right)$ functions. These results are summarized in Table 2, columns from X to XIII. The peak position of the spectra $\varepsilon_{2}^{mic}\left(\omega_{3D}\right)$ and spectra obtained using the Mie equation for the absorption coefficient *K*(*ω*) [27]:

(11) $$K\left(\omega \right)=18\left(\frac{\omega }{c}\right)\pi \varepsilon_{h}^{\frac{3}{2}}V\left(\frac{\varepsilon_{2}\left(\omega \right)}{{\left[\varepsilon_{1}\left(\omega \right)+2\varepsilon_{h}\right]}^{2}+{\left[\varepsilon_{2}\left(\omega \right)\right]}^{2}}\right),$$

where *V* = (4π/3)*R*^3^ is the volume of the spherical particle, *c* is the velocity of light and *ε_h_* is the dielectric constant of the surrounding medium, are listed in columns XIII and XII, respectively. According to Mie’s Equation (11), if *ε*_2_(*ω*) is relatively small, a resonance condition is obtained at *ε*_1_(*ω*) = −2*ε_h_*. When *ε_h_* = 1, we have Fröhlich’s condition, i.e., Re(*ε*(*ω_F_*)) = −2, where *ω_F_* is the so-called Fröhlich frequency [13]. In Equation (9), if we replace *m* = 3, *f* = 1 and substitute $\theta_{3D}\left(\omega \right)$ to Equation (8), the function obtained $\varepsilon_{2}^{mic}\left(\omega_{3D}\right)$ is given by

(12) $$\varepsilon_{2}^{mic}=9\left(\frac{\varepsilon_{2}\left(\omega \right)}{{\left[\varepsilon_{1}\left(\omega \right)+2\varepsilon_{h}\right]}^{2}+{\left[\varepsilon_{2}\left(\omega \right)\right]}^{2}}\right),$$

which practically coincides with Equation (11) for *K*(*ω*), at least for the peak position. This is also seen in Table 2 by comparing columns XII and XIII as well as from Figure 1b. The peak position of both these functions are also in good agreement with the Fröhlich frequency, *ω_F_*, listed in column XI and the position of the localized surface plasmon on the spherical particle, defined as *ω_p_*/√3 and listed in column X [27]. These results were partially confirmed earlier for noble metal spherical nanoparticles in Refs. [11,13,37] and further confirmed with experimental data obtained for spherical nanoparticles of noble metals in colloidal solutions, as well as by experimental data for the solid colloids mentioned earlier. The small deviation seen for the value *ω_p_*/√3 could be due to the fact that EELS experimental data for the *ω_p_* value were used. This varies depending on the source, especially for alkali metals, since they oxidise rapidly when exposed to air during measurements. Note that the experimental EELS data in Table 2 are taken from Refs. [38,39,40,41,42].

### 2.2. Two-Dimensional Dielectric Confinement

Calculations of $\varepsilon_{2}^{mic}\left(\omega_{2D}\right)$ for 2D confinement, using *m* = 2 and *ε_h_* = 1 in Equation (9), are shown in Figure 1, Figure 2 and Figure 3 for selected metals and the peak positions of these spectra for all of the metals studied are summarized in Table 2, columns VI–IX. Data on the peak position, *ω_2D_*, were compared with the results of simple estimations for the surface plasmon resonance using the equation *ω_s_* = *ω_p_*/(1 + *ε_h_*)^1/2^ (or *ω_s_* = *ω_p_*/√2 at *ε_h_* = 1) [43] as well as with the peak position of functions describing these surface oscillations using Ritchie’s equation—Im(1/*ε* + 1) (see Ref. [44]). Using a thermodynamic approach and Bloch’s equation, Ritchie [44] introduced the energy-loss functions—Im[1/*ε*(*ω*)] and Im[1/(*ε*(*ω*) + 1)] via real *ε*_1_(*ω*) and imaginary *ε*_2_(*ω*) parts of the complex dielectric permittivity function *ε*(*ω*) of the bulk metal. These two equations describe the probability that fast electrons crossing the material will suffer energy losses due to volume- and surface-collective electron oscillations, respectively. Ritchie predicted that the surface (or lowered) plasma loss will happen at *ω_p_*/√2 for a thin metallic film, where *ω_p_* is obtained from EELS experiments to determine the volume plasma frequency. Two types of energy losses at the plasma frequency, or volume plasma frequency, and, at a lower frequency, the surface plasmon frequency, were initially observed for many metals using EELS [38,39,40,41,42]. Later, it was predicted by Ferrell [45] that plasma oscillations can also be observed using other optical techniques, such as transmission, reflection, emission, etc., under specific experimental conditions, the next section has more details on this. The function Im[1/*ε*(*ω*)], corresponding to bulk plasma oscillations, will also be discussed in the next section.

It can be shown that the probability of transition between the vibrational energy levels described in our approach by the function $B\left(\omega_{2D}\right)\approx \varepsilon_{2}^{mic}\left(\omega_{2D}\right)=4\varepsilon_{2}\left(\omega \right)/\left[{\left(\varepsilon_{1}\left(\omega \right)+1\right)}^{2}+{\left(\varepsilon_{2}\left(\omega \right)\right)}^{2}\right]$ up to a constant factor coincides with the function Im[1/(*ε*(*ω*) + 1)], introduced in [44]. Therefore, the maxima for these two functions will be the same; see, for example, the data shown for Cs in Figure 1b. Therefore, these values are listed in Table 2 in one column only, viz column IX. Data obtained for *ω_2D_* were also compared with data from EELS and other experiments, as shown in Table 3 for some metals. Note that Table 3 was created based on a Table published in an excellent review article by Steinmann, (Ref. [46]). We have updated Steinmann’s Table with specific figures available for the metals investigated here and also with the addition of recent data from EELS and other optical experiments. Again, we see excellent correspondence between the spectral data and data obtained from simple calculations and experiments for all the metals studied here for 2D confinement, or for surface plasmon resonance, by comparing the results presented in Table 2 and Table 3.

### 2.3. One-Dimensional Dielectric Confinement

The functions $\varepsilon_{2}^{mic}\left(\omega_{1D}\right)$ for 1D confinement, calculated for metals using *m* = 1 and *ε_h_* = 1 in Equation (9), are shown in Figure 1, Figure 2 and Figure 3 for selected metals. The peak positions of these functions are summarized in column V of Table 2. In this case, the function $B\left(\omega_{1D}\right)\approx \varepsilon_{2}^{mic}\left(\omega_{1D}\right)=\varepsilon_{2}\left(\omega \right)/\left[{\left(\varepsilon_{1}\left(\omega \right)\right)}^{2}+{\left(\varepsilon_{2}\left(\omega \right)\right)}^{2}\right]$ and corresponds to the function Im[1/*ε*(*ω*)] introduced by Ritchie in 1957 [44] and earlier by Fröhlich [47] and then by Wilson [48] for describing the energy loss function due to induced volume collective electron oscillations. Peak positions for both functions ($\varepsilon_{2}^{mic}\left(\omega_{1D}\right)$ and Im[1/*ε*(*ω*)]), as well as the frequency corresponding to *ε*_1_(*ω*) = 0, are listed in columns V and IV, respectively. In Table 2, column III, experimental results obtained from EELS measurements are shown. 

Spectral frequencies corresponding to 1D confinement $\varepsilon_{2}^{mic}\left(\omega_{1D}\right)$, when *Re*ε(ν) = 0 [47,48] are near the plasma frequencies of the metals, measured using EELS initially [38,39,40,41,42] and listed in columns III–IV of Table 2. In addition, the frequencies of the spectra $\varepsilon_{2}^{mic}\left(\omega_{1D}\right)$ subject to the conditions needed for the appearance of the absorption bands of longitudinal oscillations of free electrons *ω_1D_*, match the frequency of the longitudinal mode *ω_LO_* determined using the dynamic theory of lattice vibrations, as demonstrated in Ref. [37].

These results suggest that resonance absorption at the longitudinal vibration frequency can be detected in a thin metal layer with a thickness *h* << λ, when the influence of medium polarization in the oscillation direction is not present. This agrees with results published in [49], where the appearance of absorption at the oscillation frequency LO is due to conditions within a thin dielectric film at an oblique incidence of the probe beam. For metals, this corresponds to Ferrel’s modes, which are theoretically predicted in [45] and can be seen in optical experiments using spectroscopic ellipsometry [50], as well as transmission and reflection [51,52,53,54,55] methods at an oblique incidence of *p*-polarized light within a thin film. 

As mentioned earlier, a selection of simple metals was used in this study to exclude the effect of interband transitions on the peak of the spectrum $\varepsilon_{2}^{mic}\left(\nu_{1D}\right)$ in particular. For simple metals, interband transitions occur far from free electron oscillations and don’t affect either the shape or the peak position of the longitudinal phonon (or the bulk plasma peak *ω_p_*) as shown in Figure 1, Figure 2 and Figure 3. However, for noble metals, closely spaced interband transitions do have an influence on the plasma peak. For example, for silver and gold, Drude calculations of *ω_p_* using the equation *ω_p_* = (*e^2^N_e_*/*ε_0_m_c_*)^1/2^, give values around 9 eV. However, in Refs. [52,53], the LO mode at ~3.8 eV was seen in *p*-polarized reflection and transmission spectra from thin silver films when measured at an oblique angle of incidence. Calculation of silver spectra, $\varepsilon_{2}^{mic}\left(\nu_{mD}\right),$ using optical constants from Ref. [23], for 1D, 2D and 3D confinement are shown in Figure 1c and demonstrate peak positions at 3.5 eV (for *ω_F_*), at 3.7 eV (for *ω_S_*) and at 3.78eV (for *ω_p_*). These values are in good agreement with measurements from Refs. [55,56] for *ω_p_* as well as for ω*_F_* and *ω_S_* as seen from Table 3.

Finally, we would like to note that Figure 3b actually confirms the assumption made earlier by Doyle in [25] that the colloid band resonant peak is greatly influenced by the spectral dependence of the *Reε*(*ω*) function for metal. As a result, the peak of absorption occurs where the internal field factor is a maximum and, therefore, the colloid band may be considered to be a frequency dependent local field phenomenon. Furthermore, Figure 1c shows an example of comparison of $\varepsilon_{2}^{mic}\left(\omega_{mD}\right)$ and *θ_mD_*(*ω*) functions for Rb for all types of confinement, i.e., 1D, 2D and 3D. A similar correspondence between these two functions is demonstrated for all three resonances at *ω_F_*, *ω_s_* and *ω_p_*. This leads to the conclusion that all three resonance bands can be regarded as a dispersive local field phenomenon under these conditions.

In conclusion, we note that several different approaches were suggested for the description of oscillations of bulk, surface and localised surface plasmons on spherical metal particles. We can split these approaches into macroscopic and microscopic approaches using electrodynamic, thermodynamic and hydrodynamic equations. It appears that the first theoretical description of the so-called “transition radiation” was presented by Frank and Ginsburg in 1946 [56] based on a macroscopic electrodynamic approach and further developed and demonstrated experimentally using optical experiments (see, for example, [57,58,59]). This phenomenon was later described as plasma oscillations based on a thermodynamic approach using Bloch’s equations by Ritchie [44]. Alternative approaches, such as microscopic methods based on Maxwell–Garnett effective medium theory and a dipole approximation [25,60,61], as well as Ferrell’s approach based on hydrodynamics using Laplace’s equation [46], were also explored.

The dispersive local field approach used here is also based on a microscopic dipole approximation and allows a simple equation to describe all three cases discussed, using one simple equation and a different form factor for differing confinement, in Equation (9). This approach illustrates the possibility of using a molecular approach for composite systems including liquids and solutions, solid dielectrics or crystals as well as metallic systems.

## 3. Conclusions

Table 1 and Table 2 show that the peak frequencies of the $\varepsilon_{2}^{mic}\left(\omega_{3D}\right)$ spectra for 3D confinement, corresponding to absorption by spherical metal micro-regions with dimensions *d* << λ, are seen close to the Fröhlich frequency, *ω_F_*, at *Re*ε(*ω_F_*) = −2. These frequencies are also closely associated with the absorption frequencies of spherical nanoparticles with a diameter of ~4–40 nm obtained using Mie theory [27] and also correspond to the spectral dependence of the probability of the energy loss function Im[1/(*ε*(*ω*) + 2)] due to the localised surface plasmon oscillations of spherical particles introduced in [44,62]. Frequency maxima of the spectral function, calculated for oblate spheroidal particles, $\varepsilon_{2}^{mic}\left(\omega_{2D}\right)$ for 2D confinement are observed at *ε*_1_*(ω*) = −1 and correspond to the spectral dependence of the probability of the energy loss function Im[1/(*ε*(*ω*) + 1)] due to induced surface-collective electron oscillations or to the surface plasmon as introduced in [44] and discussed in many theoretical papers, for example [63,64,65,66]. Furthermore, spectral frequencies corresponding to one-dimensional confinement $\varepsilon_{2}^{mic}\left(\nu_{1D}\right)$ at which the value of *Re*ε(*ν*) = 0 are near the plasma frequencies of the metals observed using EELS and various optical experiments. These frequencies coincide with the peak of the function Im[1/*ε*(*ω*)] introduced to describe the plasma energy loss function in [44,47,48]. Finally, we believe that this study on the effect of the local field on plasmons in simple metals may have significance in the area of Surface Enhanced Raman Spectroscopy (SERS). Strong coupling between plasmons and molecular excitons, specifically dye excitons, during SERS measurements using noble metal nanoparticles, has been observed recently, and this is being actively investigated. More details are available online in an excellent review [67]. 

## Figures and Tables

**Table 1:** Peak positions (eV) of optical and energy loss spectra for small metal particle (at *a* << λ) colloids.

Solid Colloids	ω_max_(eV) of ε_2_^mic^(ω) Spectra, Calculations			ω*_l_*, eV Opt. Transm.		ω*_l_*, eV [30,31]			ω*_l_*, eV [35]	ω*_l_*, eV EELS exp. [29]		ε_∞_ [24]
[30]	3D	2D	1D	[25,33].		Calculat.		Exp.	Exp.	1^st^ peak ω_s_	2^nd^ peak ω_p_	ε*_h_*
				Exp.	Calc	*l* = 1;	*l* = ∞					
I	II	III	IV	V		VI		VII	VIII	IX	X	XI
Na+NaCl	2.28	3.01	5.9	2.23	2.43	2.39		2.2	2.2			2.48
				2.35 [26]	2.2							
Na+NaBr	2.25	2.95	5.9	2.1		2.25;	2.95		2.11	2.2	5.7	2.65
Na+NaI	2.15	2.86	5.9			2.15;	2.85		2.07	2.3	5.7	2.96
K+KF	1.8	2.33	3.8	1.89		1.8;	2.25			2.3	3.4	1.89
K+KCl	1.67	2.17	3.8	1.7;	1.72	1.7;	2.09 ^+^	1.6	1.7			2.32
K+KBr	1.64	2.13	3.8	1.61;	1.65	1.63;	2.05 ^+^	1.46	1.68			2.44
K+KI	1.58	2.06	3.8	1.4;	1.55	1.55;	1.99 ^+^	1.4	1.54–1.46			2.66
Rb+RbF	1.6	2.05	3.4			1.54;	1.98 ^+^			2.1	3.4	1.94
Rb+RbI	1.42	1.84	3.36			1.36;	1.79 ^+^		1.52-1.35			2.63
CsBr	1.2	1.6	3.08			1.12;	4.05 ^+^		1.18			2.83
LiF	2.1	2.75	6.9						2.76-2.54			2.04
LiH	2.1	2.75	6.9	1.91 [33]		2.4;	3.21 ^+^					3.6
	2.02	2.66	6.9			2.3;	3.1 ^+^					3.94
Al+Al_2_O_3_	8.9	10.9	15.03			8.8;	10.8			10.3 [39,41]	15.3 [38,39]	1
	5.3		15.03					5.14				3.72

^+^ Data calculated in this work.

**Table 2:** Calculated and experimental peak positions of *ε*_2_*^mic^*(*ω_mD_*) spectra calculated for 1D (*m* = 1), 2D (*m* = 2) and 3D (*m* = 3) confinement and experimental data for the bulk (*ω_p_*) and surface (*ω_s_*) plasmons.

Metal	ε_∞_	1D—ω_p_, eV			$2D—\omega_{S}\;=\;\omega_{p}/\sqrt{2},\;\mathrm{eV}$				$3D—\omega_{F}=\;\omega_{p}/\sqrt{3},\;\mathrm{eV}$			
		EELS ω_p_, eV	ω_p_ at ε_1_(ω) = 0	ε_2_^mic^(ω_1D_) = Im[1/ε(ω)]	Exper. EELS	ω_p_/√2	ω_S_ at ε_1_(ω) = −1	ε_2_^mic^(ω_2D_) = Im[1/ε + 1]	ω_p_/√3	ω_F_ at ε_1_(ω) = −2	Mie Eq.(11)	ε_2_^mic^(ω_3D_) = Im[1/ε(ω)]
I	II	III	IV	V	VI	VII	VIII	IX	X	XI	XII	XIII
Li	1.02	7.1 ^a^ 8.2 ^c^	6.7	7	4.7 ^a^ 4.28 ^d^	5.04	4.64	4.83	4.1	3.5	3.65	3.56
Na	1.06	5.7 ^a^ 5.4 ^c^	6	5.92	3.98 ^d^	4.04	4	3.97	3.3	3.3	3.3	3.29
K	1.06	3.8 ^c^	3.9	3.87	2.73 ^d^	2.6	2.85	2.84	2.15	2.3	2.29	2.28
Rb	1	3.41 ^a^	3.36	3.34	2.46 ^d^	2.43	2.5	2.52	1.99	2.02	2.04	2.03
Cs	1	2.9 ^a^	3.07	3.08	1.99 ^d^	2.43	2.2	2.2	1.98	1.8	1.81	1.81
Al	1.11	15.0 ^a^ 15.3 ^b^	15.1	15.04	10.3 ^d^ 10.3 ^b^	10.8	11	10.9	8.8	8.9	8.87	8.94
Be	1.02	18.7 ^a^ 18.4 ^b^	17.5	18.1	11.9 ^b^	13.1	12.2	12.5	10.7	9.9	10.55	10.4
Mg	1.01	10.3 ^a^ 10.6 ^b^	10.8	10.7	7.38 ^d^ 7.1 ^b^	7.5	8.3	8.27	6.12	6.3	6.3	6.3
Ga	1.0	13.8 ^a^	14.1	14.07	10.2 ^e^	9.8	10.1	10.2	8.03	8.3	8.33	8.4
In	1.0	11.4 ^a^ 11.3 ^c^	11.44	11.43	8.7 ^e^	8.13	9.0	8.92	6.6	7.4	7.52	7.4
Sn	1.203	13.7 ^a^ 14.3 ^b^	13.0	13.2	10.5 ^b^	9.5	9.0	9.2	7.6	7.3	7.7	7.54
Si		16.7 ^a^ 17.0 ^c^	16.3	16.7	11 ^a^	12	11.7	11.8	9.8	9.8	10	9.97

^a^—Ref. [38], ^b^—Ref. [39], ^c^—Ref. [40], ^d^—Ref. [41], ^e^—Ref. [42].

**Table 3:** Experimental data obtained from EELS and optical measurements for bulk and surface plasmons for simple metals.

Plasmon Type	N	Effect	References From [46]	Optical Spectra, Peak Position, eV	EELS Data Bulk Surface (SPR)		Calculation ω*_l_*, eV
Radiative	1	Plasma radiation	Ag: [34,35,36,37,38,39] Al: [43,44,45]	Ag: 3.54–3.75 Al: 15.2–15.5	15.3 [39,42]	10.3 [39,42]	15.9 [39]
Radiative	2	Optical plasma Resonance in transmission	Ag: [40,59,60,89] K: [12,61,72] Al: [62,63,66,67] Mg: [86]	Ag: 3.75–3.8 K: 3.54–3.82 Al: 14.8–14.9 Mg: 10.1	3.8 [40] 10.5 *; 10.6 [39]	7.1 [39]	10.9; 7.7 [39]
Radiative	3	Optical plasma Resonance in reflection	Al: [90,91]	Al: 15.3			
Radiative	4	Optical plasma Resonance in photoemission	K–Cs: [70,71,72] Al: [66,67,73,91,92]	K–3.7; Na–5.9; Rb–3.1; Cs–2.87; Al: 14.85–14.9	Li—9.5; Na—5.4 [40]; K—3.8 [40]; Cs—2.9 [41]; Rb—3.41 [38]	Na—3.8 [40]; Rb—2.46 [42]; Cs—1.99 [42]	Li—8.0; 5.7 [39]; Li—8.1; Na—6.0 [40]; K—4.4; Rb—4.0 [42]; Cs—3.6 [40]
Radiative	5	Plasma radiation excited by light	Ag: [75,76,77,78,83] K: [79,87]	Ag: 3.77–3.8 K: 3.76			Ag—DEF, this work 3.5, 3.7, 3.78
Non-Radiative	6	Frustrated total Reflection	Ag: [99]	Ag: 3.6			
Non-Radiative	7	Radiative decay of tangential Surface plasmon	Ag: [27,28,29,50,51,53,93] Al: [53,100]	Ag: 3.49–3.82 Al: 9.7 (2D)	15 [40]	10.3 [41]	

* Data from Schmüser, P.Z. Physik 1964, 180, 105.
